# Mechanisms, Efficacy, and Clinical Applications of Platelet-Rich Plasma in Tendinopathy: A Comprehensive Review

**DOI:** 10.7759/cureus.65636

**Published:** 2024-07-29

**Authors:** Prathamesh Kale, Hardik Patel, Ankit M Jaiswal

**Affiliations:** 1 Orthopaedic Surgery, Jawaharlal Nehru Medical College, Datta Meghe Institute of Higher Education and Research, Wardha, IND

**Keywords:** injection techniques, clinical efficacy, growth factors, regenerative medicine, prp, tendinopathy

## Abstract

Tendinopathy poses a significant clinical challenge characterized by chronic tendon pain, swelling, and impaired function, affecting athletes and the general population. Current treatments often provide limited success, necessitating exploration into regenerative therapies such as platelet-rich plasma (PRP). PRP harnesses the regenerative potential of autologous platelets and growth factors to promote tendon healing. This review aims to comprehensively examine the mechanisms, efficacy, and clinical applications of PRP in tendinopathy. We discuss the pathophysiology of tendinopathy, highlighting collagen disorganization, increased ground substance, and inflammatory changes.

PRP’s action mechanism involves releasing bioactive molecules that stimulate cellular proliferation, collagen synthesis, and tissue remodeling. Clinical studies and trials evaluating PRP in various tendinopathies, including Achilles, patellar, and rotator cuff tendinopathy, are reviewed to assess its efficacy and effectiveness compared to traditional therapies. Practical aspects, such as preparation methods, injection techniques, and safety considerations, are discussed to provide insights into optimal PRP administration. Challenges, including protocol variability and evidence gaps, are addressed, and future research and clinical practice directions are proposed. By synthesizing current knowledge, this review aims to guide clinicians in enhancing treatment strategies and advancing the field of tendon regenerative medicine.

## Introduction and background

Tendinopathy is a spectrum of tendon disorders manifesting through pain, swelling, and impaired functionality. These conditions are typically chronic, profoundly impacting an individual's quality of life by hindering daily activities and athletic pursuits [1]. Tendinopathy frequently affects tendons such as the Achilles, patellar, rotator cuff, and lateral epicondyle. The underlying pathophysiology is complex and multifactorial, involving mechanical overloading, microtrauma, and a subsequent failed healing response [2]. Histological changes in tendinopathy include collagen disorganization, increased ground substance, neovascularization, and inflammatory cells. These alterations contribute to tendon degeneration and pain, underscoring the need for effective treatment strategies to restore function and alleviate symptoms [3].

Platelet-rich plasma (PRP) is an autologous blood product rich in platelets, which are key to releasing various growth factors and cytokines that facilitate tissue repair and regeneration. PRP is produced by centrifuging the patient's blood to concentrate platelets and other bioactive molecules [4]. PRP is injected into the affected tendon or other tissues to promote healing. The rationale for PRP therapy lies in the high concentration of growth factors, such as platelet-derived growth factor (PDGF), transforming growth factor-beta (TGF-β), and vascular endothelial growth factor (VEGF), which are believed to enhance the body’s natural healing processes. PRP has gained popularity in recent years as a promising treatment option for various musculoskeletal conditions, including tendinopathy, due to its potential to improve tendon healing and reduce pain [5].

Treating tendinopathy is particularly challenging due to the chronic and degenerative nature of the condition. Traditional therapies, such as physical therapy, nonsteroidal anti-inflammatory drugs (NSAIDs), and corticosteroid injections, often provide only temporary relief and may not address the underlying pathology [6]. PRP offers an alternative approach by targeting the biological mechanisms of tendon healing, aiming to enhance tissue repair, reduce inflammation, and improve tendon function. Its minimally invasive nature and potential for long-term healing make PRP an attractive option for patients and clinicians. Despite its growing popularity, the efficacy of PRP remains a subject of ongoing research and debate, underscoring the need for a comprehensive review of the current evidence [7].

This review aims to provide a thorough and up-to-date analysis of PRP in the treatment of tendinopathy. Specifically, the objectives are to summarize the current understanding of tendinopathy, including its pathophysiology and clinical presentation; elucidate the mechanisms by which PRP promotes tendon healing and regeneration; critically evaluate the efficacy of PRP in treating various types of tendinopathy based on clinical studies and trials; discuss the practical aspects of PRP application, including injection techniques and protocols; examine the safety profile of PRP and potential adverse effects; identify the challenges and limitations associated with PRP therapy; and explore future directions and emerging trends in PRP use for tendinopathy. We believe this review will provide valuable insights into the role of PRP in tendinopathy management, assisting clinicians in making informed decisions and guiding future research efforts.

## Review

Understanding tendinopathy

Tendinopathy is a comprehensive term encompassing any tendon disorder resulting in pain, swelling, and impaired function. This term is more precise than the older term "tendinitis," which specifically denotes tendon inflammation. Tendinopathy can involve tendinosis, the degeneration of tendon tissue, rather than mere inflammation [2]. The exact underlying pathophysiology of tendinopathy remains incompletely understood, although it often arises from repetitive strain or overuse of the tendon. Such repetitive stress can cause microscopic tears and gradual tendon degradation over time. Other potential causes include acute injury, certain medical conditions like arthritis or diabetes, and improper technique or equipment during physical activities [2].

Clinical manifestations of tendinopathy typically feature pain, particularly with the movement of the affected joint. Patients may also experience swelling, stiffness, and diminished mobility or strength in the muscle-tendon unit. Diagnosis generally relies on the patient's history and findings from physical examination. Imaging tests such as ultrasound or MRI may be employed to corroborate the diagnosis and eliminate other potential causes [8]. In most cases, a crucial distinction between tendinopathy and tendinitis is the absence of significant inflammation. Instead, tendinopathy is more characterized by degenerative changes within the tendon itself. This underlying pathophysiology is essential for directing appropriate treatment strategies for patients with tendon disorders [8].

PRP: an overview

PRP is a concentrated plasma protein derived from the patient's blood, containing a significantly higher concentration of platelets (typically three to five times higher than baseline). This autologous treatment is rich in growth factors and cytokines, including platelet-derived growth factor, transforming growth factor-beta, vascular endothelial growth factor, epidermal growth factor, and hepatocyte growth factor (HGF) [9]. PRP exists in two main types based on leukocyte content: leukocyte-rich PRP (L-PRP), which includes a high concentration of white blood cells, and leukocyte-poor PRP (P-PRP), which contains fewer white blood cells.

The preparation of PRP involves drawing blood from the patient, centrifuging it to separate the plasma and platelets from red and white blood cells, collecting the platelet-rich plasma layer, and then injecting it into the injured area under ultrasound guidance. Although specific protocols may vary, the fundamental goal is to concentrate platelets to therapeutic levels [10]. Upon activation, the concentrated platelets in PRP release growth factors that stimulate cell proliferation, enhance angiogenesis, reduce inflammation, and promote tissue regeneration. These mechanisms are believed to accelerate the natural healing processes in damaged tissues. PRP represents a regenerative therapy that harnesses the body's innate healing capabilities by concentrating platelets and growth factors. While promising, ongoing research continues to evaluate its efficacy and optimal applications [11].

Mechanisms of PRP in tendinopathy

Role of Growth Factors and Cytokines

Growth factors and cytokines play critical roles in tendon healing and tendinopathy through anabolic and anti-inflammatory effects. On the anabolic side, growth factors such as IGF-1, TGF-β, and PDGF stimulate tenocyte proliferation, increase collagen production, and promote tenocyte differentiation. These growth factors also enhance the expression of key tendon-related genes like collagen types I and III, scleraxis, and tenascin-C, thereby facilitating regeneration and repair of the tendon matrix [12]. Regarding anti-inflammatory effects, cytokines like HGF released from PRP possess potent anti-inflammatory properties. This modulation of inflammation is crucial in managing the inflammatory environment often associated with degenerative tendinopathies, thereby supporting effective tendon healing [13]. Animal studies have additionally shown that certain growth factors, such as BMP-12 and BMP-13, can expedite tendon wound healing by enhancing blood vessel density and promoting the maturation of the tendon callus. Similarly, PDGF enhances fibroblast chemotaxis, proliferation rates, and collagen synthesis, all contributing to improved tendon repair processes [14].

PRP's Impact on Inflammation and Healing Processes

PRP has demonstrated dual benefits in treating tendinopathy, with anti-inflammatory and pro-healing effects. The anti-inflammatory properties of PRP are well-documented, as it effectively suppresses tendon cell inflammation by upregulating genes like HGF and attenuating pathways such as NF-κB signaling in macrophages and fibroblasts. PRP treatment also inhibits TNFα-induced IFNα/β and IFNγ signaling, which play roles in inflammatory conditions. These actions reduce pain associated with inflammation in acute injuries and chronic tendinopathy [15]. Beyond its anti-inflammatory effects, PRP promotes tendon healing through several mechanisms. Growth factors present in PRP, such as PDGF, enhance the chemotaxis of fibroblasts, increase their proliferation rate, and stimulate collagen synthesis, all crucial for tendon repair.

Animal studies have shown that injecting PRP into injured tendons improves tendon callus strength, stiffness, and maturation compared to controls. PRP also stimulates tenocyte proliferation, boosts collagen production, and induces tenocyte differentiation, further supporting tendon regeneration [16]. However, the effects of PRP can vary significantly. Some studies have indicated that PRP may not consistently outperform control treatments in inhibiting inflammation or promoting healing. The efficacy of PRP appears to hinge on factors like the specific preparation method of PRP and individual patient characteristics. Optimal patient selection and careful refinement of PRP preparation techniques may be necessary to fully harness its therapeutic potential in managing tendinopathy [17].

Effects on Cellular Proliferation and Differentiation

PRP significantly affects cellular proliferation and differentiation, particularly in mesenchymal stem cells and fibroblasts. Studies indicate that low concentrations of PRP, ranging from 2.5% to 10%, are optimal for promoting the proliferation of these cell types. For instance, research has shown that 5% PRP is particularly effective in enhancing undifferentiated fibroblast proliferation, with a notable increase observed as early as day three compared to lower concentrations. The proliferative effects of PRP are largely attributed to growth factors such as PDGF, as evidenced by studies where neutralizing PDGF partially inhibited PRP's mitogenic effects on muscle-derived progenitor cells [18].

In addition to its proliferative impact, PRP enhances the differentiation of mesenchymal stem cells and fibroblasts. For example, 10% PRP promotes odontogenic differentiation in neural crest-derived stem cells, significantly upregulating the expression of odontogenic genes like DSPP and BMP4. Moreover, PRP has been shown to increase alkaline phosphatase activity and promote mineralized nodule formation in mesenchymal lineage cells such as dental pulp cells, indicating enhanced osteogenic differentiation [19]. A synergistic action of various growth factors, including PDGF, TGF-β, and VEGF likely mediates these dual effects of PRP on proliferation and differentiation. However, further research is necessary to understand the underlying mechanisms fully and to optimize PRP preparation protocols for different clinical applications [9].

PRP and Collagen Synthesis

PRP has been extensively researched for its profound effects on collagen synthesis, particularly in wound healing and tissue regeneration. The mechanisms through which PRP influences collagen synthesis are diverse and crucial for tissue repair and regeneration. One primary mechanism involves stimulating type I collagen production, essential for tissue structural integrity. Studies have demonstrated that PRP significantly increases type I collagen production, as evidenced by research showing enhanced collagen synthesis in rat models following femoral bone-implant insertion compared to controls [20]. PRP also plays a pivotal role in stimulating the proliferation and differentiation of fibroblasts, key cells responsible for collagen production. Research consistently shows that PRP enhances fibroblast proliferation and collagen synthesis in various tissues, including skin and tendons [20]. These effects have wide-ranging applications, including aesthetic procedures where PRP improves skin elasticity, stimulates hyaluronic acid synthesis, and increases collagen production.

Clinical studies have supported PRP's ability to reduce wrinkles, enhance skin tone, and improve skin firmness [21]. Beyond aesthetics, PRP's capacity to stimulate collagen synthesis is crucial for wound healing across diverse tissue types such as skin, tendons, and bone. Studies have highlighted PRP's effectiveness in promoting the healing of bone implants by enhancing vascularization and improving the maturation of tendon calluses [20]. Key growth factors present in PRP, such as PDGF and HGF, play integral roles in these processes. PDGF contributes to PRP's mitogenic and proliferative effects, stimulating fibroblast proliferation and collagen synthesis. HGF, known for its anti-inflammatory properties, aids in reducing inflammation and promoting tissue repair, thereby supporting collagen synthesis and tissue regeneration [22].

Efficacy of PRP in Tendinopathy

Basic science studies have illuminated several potential mechanisms PRP could benefit from treating tendinopathy. PRP exhibits anabolic effects by stimulating the proliferation of tendon cells and increasing collagen production, which is critical for the structural integrity of tendons. Additionally, PRP possesses anti-inflammatory properties due to growth factors like HGF, which can mitigate inflammation associated with tendinopathy. Animal studies have corroborated these findings by demonstrating that PRP injections improve tendon healing, enhancing strength and maturation [23]. Despite promising results from laboratory and animal studies, the clinical efficacy of PRP for tendinopathy remains a topic of debate. While many clinical studies have reported improvements in pain and function following PRP injections for various tendinopathies, systematic reviews have highlighted inconsistencies in the evidence. For instance, a 2020 review identified multiple randomized clinical trials focused on Achilles tendinopathy, where only a small proportion showed statistically significant improvements in clinical outcomes compared to placebo or control groups [24].

**Figure 1:**
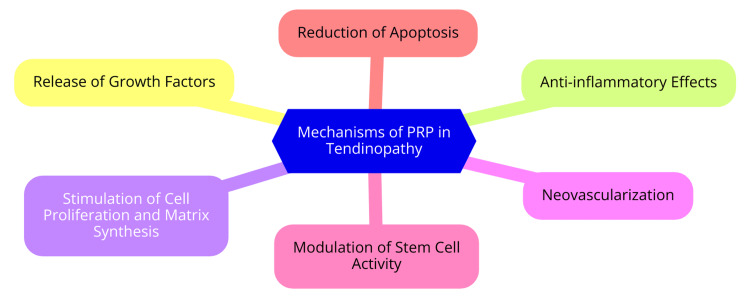
Mechanisms of PRP in tendinopathy PRP: platelet-rich plasma Image credit: Dr. Prathamesh Kale

Recent randomized trials, such as one investigating chronic midportion Achilles tendinopathy, found no significant difference in functional scores between PRP and sham injections at the six-month follow-up [24]. Several other studies and meta-analyses have similarly indicated that PRP therapy may not consistently offer superior outcomes compared to conventional treatments or placebo in managing tendinopathy [24]. In clinical practice, PRP is typically administered via ultrasound-guided intratendinous injections, often complemented by rehabilitation protocols. However, the optimal PRP preparation methods, injection techniques, and dosage regimens still need to be investigated, lacking clear consensus in the medical community. Despite the inconclusive evidence on its efficacy, PRP is generally regarded as a safe treatment option with a low risk of adverse effects relative to alternatives like corticosteroid injections [25]. Mechanisms of PRP in tendinopathy are shown in Figure 1.

Clinical applications of PRP in tendinopathy

Common Sites for PRP Application

PRP has emerged as a treatment option for various tendinopathies, including Achilles tendinopathy, patellar tendinopathy, rotator cuff tendinopathy, and lateral epicondylitis (tennis elbow). In Achilles tendinopathy, PRP is administered via ultrasound-guided intratendinous injections into the affected area of the Achilles tendon. Research, such as a study published in the Journal of Orthopaedic Research, has shown that PRP injections can significantly improve pain and function in patients with chronic Achilles tendinopathy compared to placebo injections [25]. Similarly, PRP has been utilized to treat patellar tendinopathy, where studies have indicated improvements in pain and function. The optimal PRP preparation and injection technique for patellar tendinopathy is still under investigation. For instance, research published in the Journal of Sports Sciences suggests that combining PRP injections with a rehabilitation program may reduce pain and improve function more effectively than rehabilitation alone [26].

Rotator cuff tendinopathy has also been a target for PRP treatment, with evidence suggesting that PRP injections can enhance tendon healing and alleviate pain. These injections are administered directly into the affected rotator cuff tendon under ultrasound guidance. A study in the Journal of Shoulder and Elbow Surgery demonstrated significant improvements in pain and function with PRP compared to placebo injections [27]. Furthermore, PRP has shown promise in treating lateral epicondylitis, where it is commonly injected into the common extensor tendon origin at the lateral epicondyle. Research published in the Journal of Hand Surgery has reported superior outcomes with PRP injections compared to corticosteroid injections in reducing pain and improving function in patients with lateral epicondylitis [28].

Injection Techniques and Protocols

PRP injections for tendinopathy are typically administered under ultrasound guidance to ensure precise placement into the affected tendon. Before injection, the injection site is meticulously cleaned with an antiseptic solution. The volume of PRP injected is usually small, typically ranging from 3-6 mL. A small-gauge needle injects PRP directly into the affected tendon, often employing multiple injection sites to cover the entire area of tendinopathy [25]. The optimal number of PRP injections remains debated. Some protocols advocate for a single injection, while others recommend a series of two to three injections spaced four to six weeks apart. When multiple injections are administered, they are typically spaced at intervals of four to six weeks.

PRP treatment is often complemented by a rehabilitation program that includes eccentric exercises to enhance therapeutic outcomes. Patients are commonly advised to refrain from using anti-inflammatory medications for two to three weeks before PRP injections, as these medications may interfere with the intended biological effects [29]. The composition of PRP preparation, including the concentration of platelets and growth factors, varies based on the centrifugation protocol used. Most studies utilize autologous PRP derived from the patient's blood, as allogeneic PRP from donor blood poses a potential risk of immune reactions. Another factor influencing PRP efficacy is the presence of leukocytes (white blood cells) in the PRP preparation [10]. These variables underscore the ongoing exploration and refinement of PRP protocols in treating tendinopathies.

Safety and adverse effects

Potential Risks and Side Effects

PRP injections are generally considered safe; however, like any medical procedure, they carry potential risks and side effects. The most common side effects include temporary pain or discomfort at the injection site, along with swelling, bruising, redness, and skin irritation. These effects resolve independently within a few hours or days [30]. Although rare, there is a slight risk of infection associated with PRP injections. This risk can be minimized by adhering to sterile techniques and ensuring thorough cleaning and disinfection of the injection site. Additionally, there is a very small possibility of an allergic reaction to PRP components, particularly in patients with known allergies [11]. Less common but more serious risks include nerve damage and tissue damage. Nerve damage may occur if the needle is placed too close to a nerve, resulting in symptoms like numbness, tingling, weakness, or loss of sensation. Tissue damage can result from improper injection technique or placement, potentially causing harm to surrounding tissues. To mitigate these risks, practitioners often employ image guidance and adhere to precise injection protocols [31].

Comparison with Other Treatments in Terms of Safety

PRP injections have demonstrated a favorable safety profile compared to several common treatments for tendinopathy. Corticosteroid injections, a primary alternative, pose higher risks of adverse effects such as tendon weakening, skin discoloration, infection, and potentially tendon rupture with repeated use. In contrast, PRP injections carry a lower risk of adverse effects, with mild pain, swelling, and bruising at the injection site being the most common and generally temporary side effects [32]. Hyaluronic acid injections, another common treatment, show comparable safety profiles to PRP injections. Both treatments typically result in mild and transient pain, swelling, and bruising at the injection site, suggesting PRP as a safe alternative to hyaluronic acid for managing tendinopathy [32].

Oral anti-inflammatory medications like NSAIDs, although widely used, come with systemic risks such as gastrointestinal bleeding and kidney problems. PRP injections, being a localized treatment, bypass these systemic risks, making them a safer alternative for long-term management of tendinopathy compared to oral medications [33]. Surgery, often reserved for severe tendon injuries, carries inherent risks, including infection, nerve damage, and extended recovery times. PRP injections offer a minimally invasive outpatient treatment option, presenting a safer and more convenient alternative to surgery for many patients with tendinopathy [34].

Guidelines for Safe PRP Application

Before receiving a PRP injection, patients should take several important precautions to optimize the procedure's effectiveness and minimize risks. It's recommended to avoid corticosteroid medications for two to three weeks prior, as these can interfere with PRP's therapeutic action. NSAIDs and blood thinners should be discontinued for at least one week before the injection. Systemic steroids should be stopped one to two weeks in advance, and any prior steroid injections should have been administered at least one month prior. Adequate hydration the day before the procedure is also advised [35]. The PRP injection itself should be performed by an experienced physician, often with the assistance of imaging guidance like ultrasound, to ensure precise placement into the target area. This involves drawing the patient's blood, processing it in a centrifuge to concentrate the platelets, and preparing the PRP solution for injection [36].

After the PRP injection, patients should adhere to specific guidelines for optimal recovery. They should refrain from applying ice or heat to the injection site for the first 72 hours and avoid hot baths, saunas, alcohol, and smoking during this period. Anti-inflammatory medications should be avoided for at least four weeks post-injection. Increasing activity levels and initiating physical therapy around four to five weeks after the injection are typically recommended [37]. While PRP injections generally have a favorable safety profile compared to corticosteroid injections, potential side effects may include mild pain, swelling, bruising, and, in rare instances, infection or nerve damage. These risks can be minimized through careful adherence to sterile technique and appropriate patient selection. Following the recommended pre-, peri-and post-injection protocols is crucial for ensuring the safe and effective application of PRP therapy [37].

Challenges and limitations

Variability in PRP Preparation and Administration

The effectiveness of PRP treatments for tendinopathy can vary significantly due to differences in how PRP is prepared and administered. The PRP preparation process involves several critical steps, such as blood drawing, centrifugation, and platelet activation, each of which can be conducted using varying techniques. Factors such as the volume of blood drawn, choice of anticoagulants, centrifugation speed and duration, temperature conditions, and activators like calcium chloride or thrombin can all influence the final composition of PRP. Moreover, the type of equipment used, such as centrifuges and collection tubes, can also impact the characteristics of the PRP product. Individual patient factors such as age, sex, and overall health status further contribute to variability in PRP composition [10].

PRP can be administered via different routes, including intralesional injections, topical application, or surgical implantation, with each method requiring specific considerations. The volume of PRP injected, frequency of injections, and the use of imaging guidance (e.g., ultrasound) can vary among practitioners. The tendinopathy's anatomical location and the injury's severity also play roles in determining the optimal PRP administration protocol. These variations in PRP administration methods can result in differences in the final composition of PRP, affecting factors such as platelet concentration, growth factor levels, and leukocytes and erythrocytes. These variations, in turn, may impact the biological effects and clinical outcomes of PRP treatments for tendinopathy [25].

To address this variability and enhance the efficacy of PRP treatments, efforts are underway to standardize PRP preparation protocols and thoroughly characterize the final PRP product. Standardization involves establishing consistent methods for preparing PRP and systematically evaluating the PRP product's platelet count, growth factor levels, and cellular composition. By developing more standardized protocols and ensuring comprehensive characterization of PRP, healthcare providers can improve the consistency and effectiveness of PRP treatments for tendinopathy. Ultimately, this standardization effort aims to optimize outcomes and enhance patient care in the management of tendinopathy [38].

Lack of Standardized Protocols

The lack of standardized protocols for PRP treatments presents a significant challenge in assessing and optimizing its efficacy for tendinopathy. One of the primary issues lies in the heterogeneity of PRP preparation methods. PRP composition can vary widely depending on the specific equipment and techniques used and individual patient factors. This variability complicates comparisons across studies and limits our ability to draw definitive conclusions about PRP's therapeutic potential [39]. In addition to heterogeneous preparation methods, there needs to be more consistency in how injection protocols are reported in clinical literature. Parameters such as platelet concentration, leukocyte content, injection volume, and the number of injections administered often need to be more consistently documented.

This lack of standardized reporting makes it difficult to replicate successful protocols and determine the optimal parameters for PRP administration [10]. Moreover, the optimal PRP formulation and injection protocol for different types of tendinopathy remains to be determined. Questions persist regarding the ideal PRP composition (e.g., leukocyte-rich vs. leukocyte-poor) and the most effective injection regimen (volume, frequency of injections). With clear guidelines and comparative studies, clinicians can select the most appropriate treatment strategies, potentially leading to variable outcomes and suboptimal patient care [40]. Furthermore, the lack of standardized protocols complicates the comparison of different post-PRP injection rehabilitation strategies. Because rehabilitation protocols vary widely in reported studies, assessing the relative effectiveness of various rehabilitation approaches, such as eccentric exercises or other adjunctive therapies is challenging. This need for more comparative data further impedes efforts to optimize the comprehensive management of tendinopathy using PRP [41].

I*nconsistent Results in Clinical Studies*

Several factors contribute to the challenges in assessing the efficacy of PRP for tendinopathy. One significant issue is the heterogeneity of PRP preparations. The composition of PRP can vary widely depending on the specific preparation method, the equipment utilized, and individual patient characteristics. This variability makes it challenging to compare outcomes across different studies and draw definitive conclusions about the effectiveness of PRP treatments [9]. Another contributing factor is the inconsistent reporting of PRP protocols in clinical research. There needs to be standardized reporting regarding PRP preparation and injection protocols, leading to variations in how factors like platelet concentration, leukocyte content, injection volume, and the number of injections are documented. This inconsistency hampers efforts to replicate successful protocols and identify specific factors that may influence treatment outcomes [9].

Furthermore, many randomized controlled trials (RCTs) investigating PRP for tendinopathy have been conducted with relatively small sample sizes. While RCTs are crucial for evaluating treatment efficacy, small sample sizes can reduce statistical power, making it difficult to detect meaningful treatment effects consistently. This limitation may contribute to varying or inconclusive results in the literature [23]. Additionally, clinical studies have often employed a generalized approach where PRP from commercial kits is administered at standardized doses for various tendon injuries and patient demographics without considering individual differences such as age, gender, or disease history. However, preclinical research indicates that the optimal formulation and timing of PRP therapy may vary depending on the stage of tendon degeneration and individual patient factors. This suggests that a more personalized approach to PRP treatment may be necessary to achieve more consistent and favorable clinical outcomes [23].

Cost and Accessibility Issues

The high out-of-pocket costs associated with PRP injections for tendinopathy present a substantial barrier for many patients seeking this treatment. Most insurance providers typically do not cover PRP treatments and are often categorized as experimental or investigational. As a result, patients frequently bear the full financial burden, ranging from $500 to $2,500 per injection session. For individuals requiring multiple sessions, particularly for chronic tendinopathy, the cumulative costs can escalate significantly [42]. Geographic location further influences the cost of PRP treatments, with prices generally higher in urban areas and certain regions. Factors such as the medical facility's reputation, the equipment used, and the expertise of the administering physician also impact overall costs. This geographic variability can exacerbate access challenges, particularly for patients in rural or underserved areas who may face additional expenses and logistical difficulties associated with travel for treatment [43].

The absence of insurance coverage for PRP means that access to this potentially beneficial therapy is restricted to patients capable of covering the out-of-pocket expenses. This financial barrier disproportionately affects individuals with limited financial resources, who may be unable to afford PRP despite its potential benefits for tendinopathy. Moreover, the uneven distribution of healthcare providers offering PRP injections further complicates accessibility, with certain regions and healthcare systems having limited availability of this treatment option [11].

Future directions

Advances in PRP Technology and Preparation

Advancements in PRP technology and preparation methodologies have significantly enhanced the efficacy, reliability, and accessibility of this treatment for tendinopathy. Researchers are actively investigating and refining PRP preparation methods to determine the optimal formulations for treating different types of tendon injuries. Key considerations include using leukocyte-rich or leukocyte-poor PRP and the ideal composition tailored to specific tendon pathologies. Further studies are necessary to establish the optimal number, timing, and volume of PRP injections to maximize therapeutic benefits [44].

Technological improvements in PRP devices and preparation techniques have enhanced treatment outcomes. Automated centrifugation devices and point-of-care kits have streamlined PRP preparation, ensuring consistent and high-quality platelet concentrations. These advancements have simplified PRP preparation in clinical settings, reducing reliance on specialized laboratory equipment and improving the accessibility of PRP therapy [10]. Innovative delivery systems, such as painless PRP injections using pressurized oxygen and hydroporation, are also emerging. These methods eliminate the discomfort associated with traditional needle-based injections, potentially increasing patient acceptance and adherence to PRP therapy. This advancement is particularly beneficial for patients who may have reservations about needle injections [45].

Furthermore, proteomics-based techniques have provided deeper insights into the cellular mechanisms underlying PRP's therapeutic effects. Researchers have better understood platelet physiology, their roles in inflammation, pain modulation, and interactions with the immune system. This enhanced understanding guides the development of more targeted and effective PRP formulations to optimize treatment outcomes for patients with tendinopathy [9].

Potential for Combination Therapies

The current evidence strongly supports the idea that combining different treatment modalities can enhance outcomes for patients with tendinopathy. Research has shown that combining therapies like extracorporeal shockwave therapy (ESWT) and radial shockwave therapy (RSW) can lead to significantly improved outcomes compared to either therapy alone. For instance, a recent prospective study demonstrated better results in Achilles tendinopathy patients when ESWT and RSW were used together, leveraging their complementary mechanisms to promote tissue regeneration and neovascularization and improve collagen structure [46]. Similarly, while the efficacy of PRP alone for tendinopathy remains debated, combining PRP with other modalities, such as eccentric exercises or physical therapy, has shown promise in enhancing pain relief and functional improvements. This combined approach is often part of a multimodal conservative treatment strategy that includes rest, activity modification, anti-inflammatory medications, and physical therapy modalities as first-line management for tendinopathy [13].

However, despite these promising findings, more high-quality research is needed to establish optimal combination therapy protocols. Factors such as the specific tendon affected, the severity of the condition, and the timing of interventions may influence the effectiveness of combined approaches. Future studies should directly compare combination therapies against single-modality treatments to understand their synergistic effects better and determine which patient populations are most likely to benefit. While combination therapies show potential for enhancing outcomes in tendinopathy, continued research efforts are essential to refine treatment protocols, optimize therapeutic synergies, and establish their role in the comprehensive management of this complex musculoskeletal condition [47].

Ongoing and Future Research Directions

Ongoing and future research on PRP's use for tendinopathy will prioritize optimizing PRP formulations. Continued investigation is necessary to determine the ideal PRP preparation method, including whether leukocyte-rich or leukocyte-poor PRP is more effective for different types of tendinopathy. Since the optimal PRP composition may vary depending on the specific tendon and pathology being treated, identifying the most suitable PRP formulations for each clinical scenario is a critical area of study [48]. Improving injection protocols for PRP in tendinopathy represents another crucial future direction. Further studies are needed to establish PRP injections' best number, timing, and volume. Researchers will also explore combining PRP injections with other treatments, such as eccentric exercises, to enhance therapeutic effects and improve patient outcomes [49].

Identifying the most appropriate patient populations for PRP treatment is essential. Determining which patient characteristics and tendon pathologies are most likely to benefit from PRP will help enhance outcome consistency in clinical practice. Developing patient selection criteria based on factors like chronicity, severity, and prior treatments could assist clinicians in selecting suitable candidates for PRP therapy [39]. Unraveling the underlying mechanisms of PRP's beneficial effects on tendon healing and regeneration is another vital area for future basic science research. Gaining deeper insights into the cellular and molecular mechanisms involved could guide the development of more targeted and effective PRP formulations, potentially leading to improved clinical outcomes [17].

Emerging Trends in PRP Application for Tendinopathy

An emerging trend in using PRP for treating tendinopathy is the ongoing pursuit of optimizing PRP formulations. Researchers are actively investigating the ideal composition of PRP, focusing on finding the optimal platelet and leukocyte concentrations tailored to different types of tendinopathy. Studies indicate that simply increasing platelet counts above 1x10^6/μL may not always enhance cell proliferation; hence, a nuanced approach rather than a "more is better" strategy is being explored. Another significant trend is identifying the most suitable patient populations for PRP treatment. There is increasing recognition that PRP may yield less favorable outcomes in treating advanced, degenerated tendons than in earlier tendinopathy stages. Understanding which patient characteristics and tendon pathologies are most responsive to PRP therapy could enhance the reliability of treatment outcomes in clinical settings [50].

The integration of PRP with other treatments, particularly structured rehabilitation programs and surgical debridement, is also gaining prominence. PRP is frequently combined with eccentric exercises to optimize tendon healing and functional recovery. Moreover, combining PRP with surgical debridement of degenerated tendon regions has shown promise in improving outcomes for advanced tendinopathy cases. Comparative effectiveness research between PRP and other biological therapies, such as stem cell injections, represents another critical area of investigation. For instance, studies have suggested that adipose-derived stromal vascular fraction may lead to faster improvements in Achilles tendinopathy than PRP. This underscores the importance of evaluating and understanding the relative benefits of various biological approaches for tendon treatment [24].

## Conclusions

PRP represents a promising therapeutic option in the management of tendinopathy, offering a biologically plausible approach to enhance tendon healing and alleviate symptoms. Throughout this review, we have explored the complex pathophysiology of tendinopathy and the rationale behind PRP therapy, which leverages the regenerative potential of growth factors and cytokines released from platelets. The evidence synthesized from clinical studies and trials suggests that PRP can effectively improve tendon function and reduce pain in various tendinopathies. However, results can vary depending on factors such as preparation methods, injection techniques, and patient characteristics.

Despite the growing body of literature supporting its efficacy, challenges such as variability in treatment protocols and the need for standardized guidelines still need to be addressed. Continuing research efforts are crucial to refine PRP therapies, establish optimal treatment protocols, and clarify its role in different stages and types of tendinopathy. By addressing these challenges and harnessing the full potential of PRP, clinicians can better tailor treatments to individual patient needs, ultimately improving outcomes and advancing the field of tendon regenerative medicine.
